# A Label-Free Fluorescent DNA Calculator Based on Gold Nanoparticles for Sensitive Detection of ATP

**DOI:** 10.3390/molecules23102494

**Published:** 2018-09-29

**Authors:** Jingjing Zhang, Shizhi Zhang, Chaoqun Niu, Chen Liu, Jie Du, Yong Chen

**Affiliations:** State Key Laboratory of Marine Resource Utilization in South China Sea, College of Information Science & Technology, College of Materials and Chemical Engineering, Institute of Tropical Agriculture and Forestry, Hainan University, Haikou 570228, China; zhangjingjingaoxue@163.com (J.Z.); 13698954926@163.com (S.Z.); chaoqunniu@163.com (C.N.); chen__liu@126.com (C.L.)

**Keywords:** ATP detection, label-free fluorescence, enzyme-free, gold nanoparticles, DNA calculator

## Abstract

Herein we described a deoxyribonucleic acid (DNA) calculator for sensitive detection of the determination of adenosine triphosphate (ATP) using gold nanoparticles (GNP) and PicoGreen fluorescence dye as signal transducer, and ATP and single-stranded DNA (DNA-M′) as activators. The calculator-related performances including linearity, reaction time, logic gate, and selectivity were investigated, respectively. The results revealed that this oligonucleotide sensor was highly sensitive and selective. The detection range was 50–500 nmol/L (R^2^ = 0.99391) and the detection limit was 46.5 nmol/L. The AND DNA calculator was successfully used for the ATP detection in human urine. Compared with other methods, this DNA calculator has the characteristics of being label-free, non-enzymic, simple, and highly sensitive.

## 1. Introduction

Many diseases, such as Parkinson’s disease, ischemia, hypoxia, hypoglycemia, and some malignant tumors, are closely related to the concentration of ATP [1,2,3]. Consequently, the detection of ATP is of substantial clinical importance. Methods based on fluorescent probes possess high sensitivity and selectivity as well as good compatibility with visible-light imaging of biological systems [4]. Accordingly, considerable efforts are being made to develop fluorescent sensors for the detection of ATP.

Signal amplification techniques, such as rolling circle replication [5] and strand displacement amplification [6,7,8,9], provide an effective strategy for improving the sensitivity of various biosensors. However, these methods usually suffer from a high background signal and often rely on the use of biological enzymes and fluorescent labels. Biological enzymes and fluorescent labels are typically expensive and sensitive to temperature and pH. Compared with signal amplification, the approach of background signal suppression is often simpler and requires a shorter reaction time [10,11]. Nanoparticles such as nanoceria [12] and gold nanoparticles (GNPs) [13,14] can be used as quenchers to suppress the background signal and enhance the sensor’s sensitivity. However, most signal suppression approaches involve high cost due to fluorescence-labeled probe. To date, several label-free fluorescence methods, such as those based on the fluorescent dye probes thiazole orange [15], PicoGreen [16,17], and SYBR Green I [18], have been developed to detect ATP. However, neither signal amplification nor signal suppression was applied in most of these label-free fluorescence methods. In this study, we investigated the use of GNPs and the PicoGreen probe to construct a biosensor for ATP based on label-free fluorescence. The developed assay is simple, sensitive, and does not require enzymes and fluorescent label.

Molecular computers are very promising for applications in nanotechnology, medicine, and biotechnology. To date, certain math problems have been solved using DNA computers [19,20]. Molecular calculators can perform molecular Boolean operations. Although many molecular calculators, such as AND, INH + NINH, and INHIBIT [11,21,22], have been developed, there is still a need to construct DNA calculators that are more sensitive, simpler, and less expensive. The DNA calculator designed in this work has two different functions [23,24,25,26,27,28,29,30]. Firstly, this calculator is actually a kind of AND logic gate. Secondly, this DNA calculator can be used to sensitively determine and calculate ATP concentration based on the changes in fluorescence intensity. Hence, we constructed an AND DNA calculator based on label-free fluorescence using GNPs and PicoGreen, which is simple, enzyme-free, and highly sensitive. It is hoped that the general method developed in this work can be adopted for the detection of various different proteins and ions.

## 2. Results and Discussion

### 2.1. Establishment of AND DNA Calculator

**Scheme 1:**
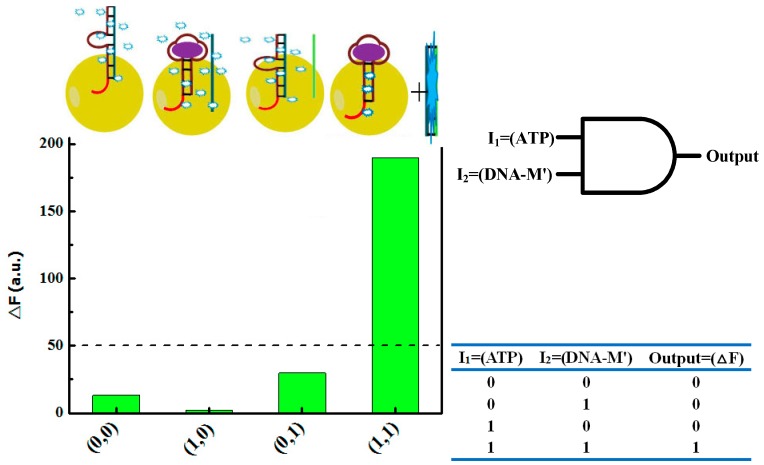

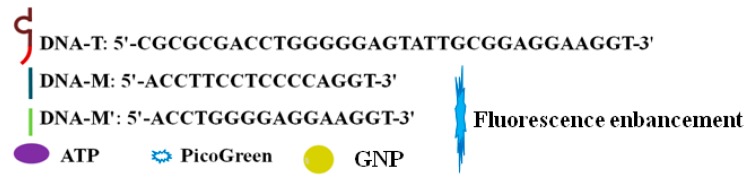
Operating principle of the AND DNA calculator.

Scheme 1 illustrates the operating principle of the GNP–PicoGreen-based AND DNA calculator for the detection of ATP. This assay exploits the properties of PicoGreen and GNPs. PicoGreen forms a highly luminescent complex upon binding dsDNA compared to binding ssDNA [16,17]. ssDNA and dsDNA (DNA-T + DNA-M) with a sticky end can adsorb onto the surfaces of GNPs at a particular ionic strength, enabling energy transfer to occur from the DNA to the GNPs and effectively quenching the fluorescence intensity [13]. However, the fully complementary dsDNA is released from the GNPs because it does not bind at the same ionic strength, and the PicoGreen fluorescence is observed. We used ssDNA (DNA-T) as an ATP-specific oligonucleotide and dsDNA (DNA-T + DNA-M) as the probe. The inputs are ATP and DNA-M′ ssDNA, and the output is the fluorescence signal from PicoGreen. The presence of ATP and DNA-M′ triggers the structural conversion of the probe from DNA-T + DNA-M dsDNA to DNA-M + DNA-M′ dsDNA and induces a change in the PicoGreen fluorescence intensity. The presence of ATP or DNA-M′ corresponds to input values of 1, whereas their absence corresponds to input values of 0. A high PicoGreen fluorescence intensity corresponds to an output value of 1, whereas a low fluorescence intensity corresponds to an output value of 0. As shown in Scheme 1, a strong output response occurs only when both ATP and DNA-M′ are present (1,1), which is the Boolean calculation for an AND logic gate.

### 2.2. Assay Feasibility

**Figure 1:**
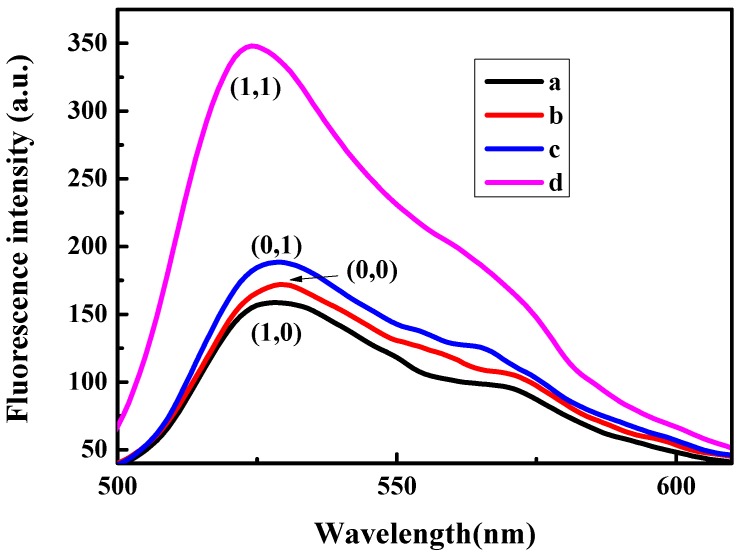
Fluorescence emission spectra: (**a**) DNA-T + DNA-M dsDNA, GNP and 200 µL of 10 µM ATP; (**b**) DNA-T + DNA-M dsDNA and GNP; (**c**) DNA-T + DNA-M dsDNA, GNP, and DNA-M′; (**d**) DNA-T + DNA-M dsDNA, GNP, DNA-M′, and 200 µL of 10 µM ATP.

Firstly, we used fluorescence spectroscopy to investigate the feasibility of the proposed assay. The differences between the peaks of the four curves and the peak of the a-curve in Figure 1 were used as the output signal, respectively. If the fluorescence intensity difference, ΔF, was greater than 50 a.u., the output signal was 1, otherwise it was 0. As shown in Figure 1, adsorption of the DNA-T + DNA-M dsDNA with sticky ends on the GNP surface (input 0,0) resulted in a strong quenching of the PicoGreen fluorescence owing to fluorescence resonance energy transfer (FRET) [13]. Upon the individual addition of ATP (1,0) or DNA-M′ ssDNA (0,1), the fluorescence intensity barely changed because the dsDNA remained adsorbed on the GNP surface through the sticky end, resulting in FRET. However, the addition of both ATP and DNA-M′ (1,1) resulted in the binding of ATP to DNA-T ssDNA and the release of DNA-M ssDNA, which subsequently bound to DNA-M′ ssDNA to form DNA-M + DNA-M′ dsDNA, causing an obvious increase in the fluorescence intensity due to binding of the PicoGreen to the DNA-M + DNA-M′ dsDNA. This increase in the PicoGreen fluorescence intensity was induced only by the dual input of ATP and DNA-M′, which conforms to the Boolean calculation of an AND logic gate as shown in Scheme 1.

**Figure 2:**
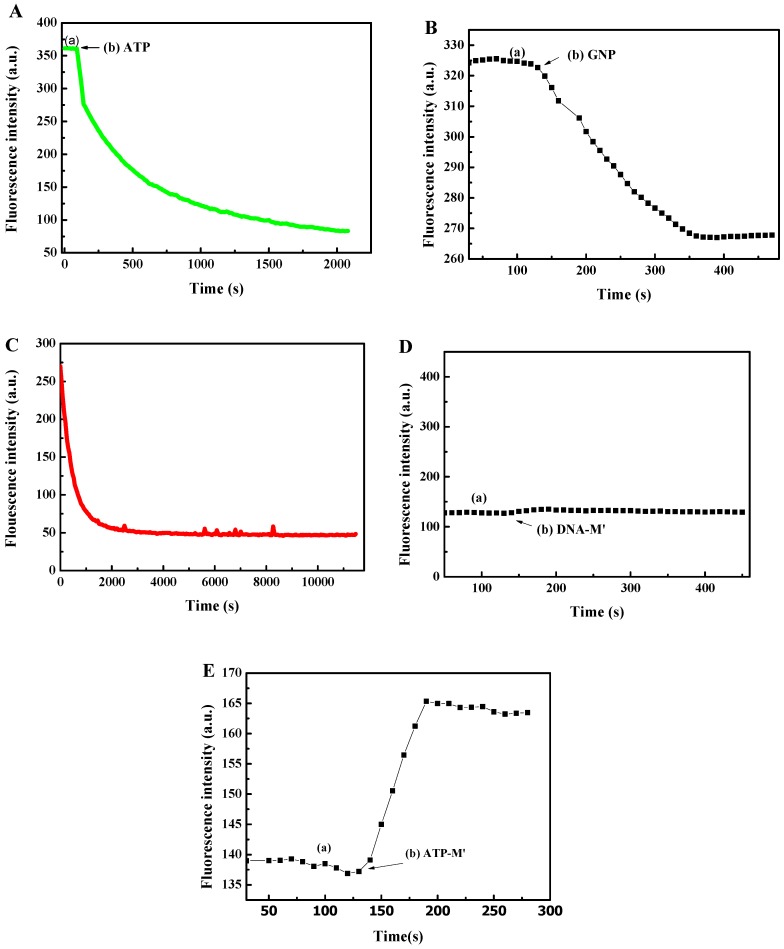
Monitoring of the reaction progress under various conditions: (**A**) (a) DNA-T + DNA-M dsDNA; (b) 200 µL of 10 µM ATP. (**B**) (a) DNA-T + DNA-M dsDNA; (b) GNP. (**C**) DNA-T + DNA-M dsDNA, GNP, and 200 µL of 10 µM ATP. (**D**) (a) DNA-T + DNA-M dsDNA and GNP; (b) DNA-M′. (**E**) (a) DNA-T + DNA-M dsDNA, GNP, and 200 µL of 10 µM ATP; (b) DNA-M′.

To further explore the feasibility of the assay principle, we monitored the reaction progress over time. Figure 2A shows that the fluorescence intensity decreased significantly when ATP was added to DNA-T + DNA-M dsDNA because the combination of ATP and DNA-T ssDNA led to the opening of the dsDNA complex [16]. Figure 2B demonstrates that the addition of GNP caused the fluorescence intensity to visibly decrease as a result of quenching [13,14]. The fluorescence intensity was weak upon the individual addition of ATP (1,0) or DNA-M′ (0,1), as shown in Figure 2C and Figure 2D, respectively. In contrast, the addition of both ATP and DNA-M′ (1,1) resulted in a clear increase in the fluorescence intensity. These results were consistent with those shown in Figure 1 and further demonstrate the feasibility of the proposed system.

### 2.3. Assay Linearity and Sensitivity

**Figure 3:**
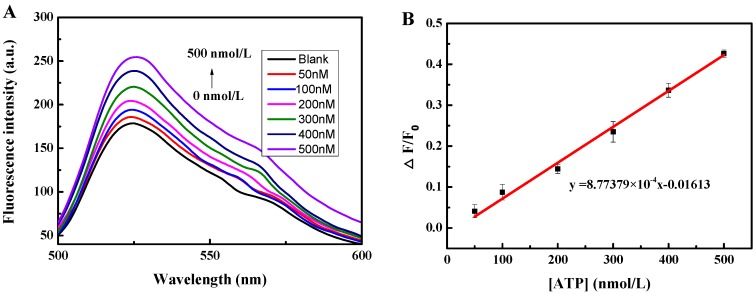
(**A**) Fluorescence emission spectra at various ATP concentrations. (**B**) Linearity for detection of ATP.

The linearity and sensitivity were further examined by acquiring fluorescence spectra at various concentrations of ATP. Figure 3 shows that increasing the concentration of ATP from 50 to 500 nmol/L led to a linear increase in the relative change of fluorescence, demonstrating that the fluorescence signal is strongly dependent on the ATP concentration. The linear regression equation was *y* = 8.77379 × 10^−4^*x* − 0.01613 (*x* and *y* refer to the ATP concentration and the relative change of fluorescence, respectively) with an R^2^ value of 0.993, and the limit of detection (LOD) was 46.5 nmol/L (3σ/slope). Therefore, within this linear range, this equation can be used to quantitatively estimate the ATP concentration. Compared with most other methods (Table 1), the results clearly show that our method affords a lower detection limit and sufficient linearity and sensitivity for the detection of the target analyte ATP.

### 2.4. Assay Selectivity

**Figure 4:**
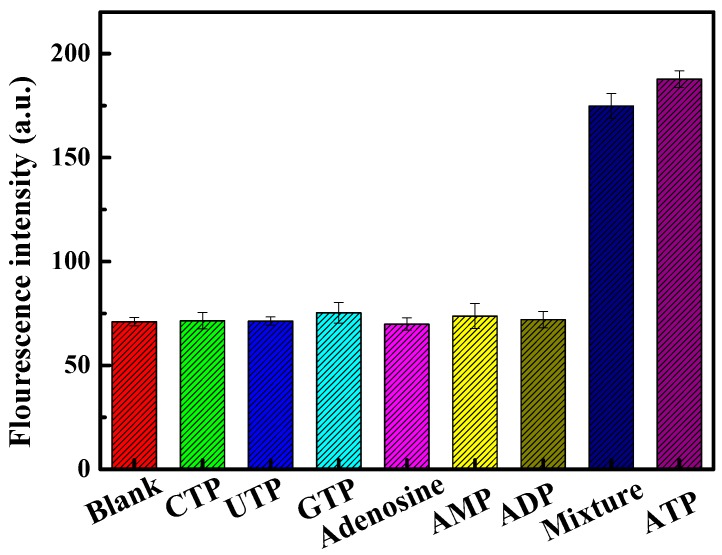
Selectivity of the assay for the detection of ATP. The mixture consists of ATP, CTP, UTP, GTP, adenosine, AMP, ADP, each in concentrations of 200 nmol/L.

To assess the selectivity of the proposed sensing system, the responses of the assay to several similar control molecules (GTP, UTP, CTP, adenosine, ADP, and AMP) were evaluated under the same experimental conditions. Figure 4 shows that only negligible changes in fluorescence intensity occurred in the presence of these small molecules compared with a blank solution. However, the fluorescence intensity increased significantly in the presence of the target molecule ATP. Furthermore, the enhancement in fluorescence intensity caused by a mixture of these small molecules (ATP, GTP, UTP, CTP, adenosine, ADP, and AMP) was similar to that caused by ATP alone. These results clearly demonstrate that the proposed assay has sufficient selectivity for the detection of ATP.

### 2.5. Determination of ATP Concentration in Real Urine Samples

To validate the viability of the proposed assay, we conducted standard addition recovery tests by adding ATP to samples of human urine and serum. As shown in Table 2, the recovery rates of urine and serum samples were 98.4 to 101.4% and 101.2 to 103.6%, respectively. The experimental results were satisfactory, indicating that the proposed method is reliable and has immense potential for determining the ATP concentration not only in real urine but also in serum samples, which endow this proposed system more significant for the practical application. 

## 3. Materials and Methods

### 3.1. Materials and Reagents

Adenosine, adenosine diphosphate (ADP), adenosine monophosphate (AMP), Cytidine triphosphate (CTP), ATP, guanosine triphosphate (GTP), uridine triphosphate (UTP), and PicoGreen dye were supplied by Shanghai Yi Sheng Technology Co., Ltd. (Shanghai, China). All oligonucleotides were purchased from Beijing Genomics Institute (Beijing, China). The DNA sequences are listed in Table 3. PicoGreen dsDNA quantitation reagent was purchased from Shanghai Yi Sheng Biotechnology Co., Ltd. (Shanghai, China). GNP was purchased from the Lianshi Mall (Beijing, China).

All reagents were diluted in buffer solution (10 mmol/L Tris, 10 mmol/L MgCl_2_, 50 mmol/L NaCl, pH 7.5). The volumes of buffer solution, PicoGreen (200-fold dilution), double-stranded DNA (dsDNA), single-stranded DNA (ssDNA), and GNPs (10-fold dilution) were 1800 µL, 30 µL, 90 µL, 100 µL, and 10 µL, respectively, in all experiments. Components in each experiments illustrated in Figure 1 and Figure 2 are listed in Table 4 and Table 5, respectively.

### 3.2. Apparatus

The fluorescence spectra and reaction progress were measured using a fluorescence spectrometer (RF-6000, Shimadzu, Tokyo, Japan). The fluorescence spectra were acquired with an excitation wavelength of 480 nm and a detection range from 495 to 700 nm. The reaction progress was monitored using an excitation wavelength of 480 nm and an emission wavelength of 520 nm.

### 3.3. ATP Detection in Real Samples

Fresh human serum samples were supplied by Nanjing China Senbeijia Biological Technology Co., Ltd., which did not do any other pretreatment. We obtained urine from a healthy adult and did not add any detergent. The urine sample was centrifuged at 12,000 rpm for 2 min and the supernatant was diluted tenfold with buffer solution. ATP was then added to the diluted urine and fresh human serum samples at concentrations of 100, 300, and 500 nmol/L, and each measurement was performed in triplicate. Finally, the ATP recovery was calculated.

## 4. Conclusions

We developed a label-free-fluorescence-based and enzyme-free AND DNA calculator for the sensitive and convenient determination of ATP concentration. This AND DNA calculator has many important features. First, the GNP-based DNA calculator partially eliminates the background signal and improves the sensitivity of the sensor. Second, this DNA calculator does not require fluorescent labeling or enzymes, leading to a lower assay cost. Third, this DNA calculator is highly selective for ATP in the presence of similar small molecules (GTP, UTP, and CTP) and affords a lower detection limit compared with most of the previously reported methods. Most importantly, this DNA calculator can be successfully applied to the detection of ATP in urine samples, demonstrating its immense potential for practical clinical diagnosis. Owing to these advantages, the proposed method could also be extended to facilitate the detection of other small molecules and proteins in a label-free and enzyme-free manner.

## Figures and Tables

**Table 1:** Other methods for ATP detection.

Methods	LOD	Linear Range	Correlation Coefficient (R^2^)	Reference
Graphene-Based Aptamer Logic Gates	2.07 μmol/L	5–50 µmol/L	0.997	23
Naphthalimide-rhodamine Compound (NR) probe	100 nmol/L	0.1–10 µmol/L	N/A	24
Oligonucleotide-templated Silver Nanoclusters	33 nmol/L	0.1–10 µmol/L	0.98	25
Core-Shell Ag@SiO_2_ Nanoflares	8 μmol/L	0–0.5 mmol/L	N/A	26
DNA/Ag Nanoclusters	91.6 nmol/L	0.5–8.0 μmol/L	0.99	27
Gold Nanoclusters	43 μmol/L	50–100 µmol/L	0.9908	28
Carbon nanoparticles-Fe^3+^	0.48 μmol/L	0.5–50 µmol/L	0.99733	29
ATP Displace Adsorbed DNA from Nanoceria	54 pmol/L	0.1 nmol/L–1.5 µmol/L	0.996	12
Magnetite Nanoparticle	30 nmol/L	100–1000 nmol/L	0.9950	30
GNP	46.5 nmol/L	50–500 nmol/L	0.99391	This work

**Table 2:** Detection of ATP concentration in the real samples of urine and serum.

Samples	ATP		Proposed	Method	
	Added	Found	Recovery	SD (*n* = 3)	RSD (*n* = 3)
	(nmol/L)	(nmol/L)	(%)	(nmol/L)	(%)
Urine-1	100.00	99.9749 97.0575 98.1570	98.4	1.2	1.2
Urine-2	300.00	295.1230 305.9749 311.0817	101.4	6.65	2.7
Urine-3	500.00	513.4384 492.3729 481.5200	99.2	13.2	2.6
Serum-1	100.00	110.8895 98.1786 101.8063	103.6	5.3	5.2
Serum-2	300.00	299.0586 301.0191 303.8109	101.3	4.1	1.3
Serum-3	500.00	506.5864 498.6701 502.3293	101.2	5.6	1.1

**Table 3:** DNA sequences.

Oligonucleotide	Sequence
DNA-T	5′-CGC GCG ACC TGG GGG AGT ATT GCG GAG GAA GGT-3′
DNA-M	5′-ACC TTC CTC CCC AGG T-3′
DNA-M′	5′-ACC TGG GGA GGA AGG T-3′

**Table 4:** Components in the experiment of Figure 1.

Name	Components
a-curve	1800 µL buffer, 30 µL PicoGreen, 90 µL DNA-T + DNA-M dsDNA, 10 µL GNP, 200 µL of 10 µM ATP
b-curve	1800 µL buffer, 30 µL PicoGreen, 90 µL DNA-T + DNA-M dsDNA and 10 µL GNP
c-curve	1800 µL buffer, 30 µL PicoGreen, 90 µL DNA-T + DNA-M dsDNA and 10 µL GNP, 100 µL DNA-M′
d-curve	1800 µL buffer, 30 µL PicoGreen, 90 µL DNA-T + DNA-M dsDNA and 10 µL GNP, 100 µL DNA-M′, 200 µL of 10 µM ATP

**Table 5:** Components in the experiment of Figure 2.

Name		Components
Figure 2A	(a)	1800 µL buffer, 30 µL PicoGreen, 90 µL DNA-T + DNA-M dsDNA
	(b)	200 µL of 10 µM ATP
Figure 2B	(a)	1800 µL buffer, 30 µL PicoGreen, 90 µL DNA-T + DNA-M dsDNA
	(b)	10 µL GNP
Figure 2C		1800 µL buffer, 30 µL PicoGreen, 90 µL DNA-T + DNA-M dsDNA, 10 µL GNP, 200 µL of 10 µM ATP
Figure 2D	(a)	1800 µL buffer, 30 µL PicoGreen, 90 µL DNA-T + DNA-M dsDNA, 10 µL GNP
	(b)	100 µL DNA-M′
Figure 2E	(a)	1800 µL buffer, 30 µL PicoGreen, 90 µL DNA-T + DNA-M dsDNA, 10 µL GNP, 200 µL of 10 µM ATP
	(b)	100 µL DNA-M′
